# Tumor-Infiltrating Lymphocytes in the Tumor Microenvironment of Laryngeal Squamous Cell Carcinoma: Systematic Review and Meta-Analysis †

**DOI:** 10.3390/biomedicines9050486

**Published:** 2021-04-28

**Authors:** Juan P. Rodrigo, Mario Sánchez-Canteli, Fernando López, Gregory T. Wolf, Juan C. Hernández-Prera, Michelle D. Williams, Stefan M. Willems, Alessandro Franchi, Andrés Coca-Pelaz, Alfio Ferlito

**Affiliations:** 1 Department of Otolaryngology, Hospital Universitario Central de Asturias-Instituto de Salud del Principado de Asturias (ISPA), 33011 Oviedo, Spain; uo216751@uniovi.es (M.S.-C.); lopezafernando@uniovi.es (F.L.); cocaandres@uniovi.es (A.C.-P.); 2 IUOPA-University of Oviedo, 33006 Oviedo, Spain; 3 CIBERONC, Instituto de Salud Carlos III, 28029 Madrid, Spain; 4 Department of Otolaryngology-Head and Neck Surgery, University of Michigan Health System, Ann Arbor, MI 48109, USA; gregwolf@med.umich.edu; 5 Department of Pathology, Moffitt Cancer Center, Tampa, FL 33612, USA; Juan.Hernandez-Prera@moffitt.org; 6 Department of Pathology, The University of Texas M. D. Anderson Cancer Center, Houston, TX 77030, USA; MDWillia@mdanderson.org; 7 Department of Pathology, University Medical Center Groningen, 9713 GZ Groningen, The Netherlands; s.m.willems@umcg.nl; 8 Department of Translational Research, School of Medicine, University of Pisa, 56126 Pisa, Italy; alessandro.franchi@unipi.it; 9 Coordinator of the International Head and Neck Scientific Group, 35125 Padua, Italy; profalfioferlito@gmail.com

**Keywords:** larynx, squamous cell carcinoma, tumor-infiltrating lymphocytes, prognosis

## Abstract

The presence of tumor-infiltrating lymphocytes (TIL) in the tumor microenvironment has been demonstrated to be of prognostic value in various cancers. In this systematic review and meta-analysis, we investigated the prognostic value of TIL in laryngeal squamous cell carcinoma (LSCC). We performed a systematic search in PubMed for publications that investigated the prognostic value of TIL in LSCC. A meta-analysis was performed including all studies assessing the association between TIL counts in hematoxylin-eosin (HE)-stained sections, for CD8+ and/or CD3+/CD4+ TIL and overall survival (OS) or disease-free survival (DFS). The pooled meta-analysis showed a favorable prognostic role for stromal TIL in HE sections for OS (HR 0.57, 95% CI 0.36–0.91, *p* = 0.02), and for DFS (HR 0.56, 95% CI 0.34–0.94, *p* = 0.03). High CD8+ TIL were associated with a prolonged OS (HR 0.62, 95% CI 0.4–0.97, *p* = 0.04) and DFS (HR 0.73, 95% CI 0.34–0.94, *p* = 0.002). High CD3+/CD4+ TIL demonstrated improved OS (HR 0.32, 95% CI 0.16–0.9, *p* = 0.03) and DFS (HR 0.23, 95% CI 0.10–0.53, *p* = 0.0005). This meta-analysis confirmed the favorable prognostic significance of TIL in LSCC. High stromal TIL evaluated in HE sections and intra-tumoral and stromal CD3+, CD4+ and/or CD8+ TIL might predict a better clinical outcome.

## 1. Introduction

Head and neck squamous cell carcinoma (HNSCC) is the most frequent malignancy in the head and neck region, and the seventh most commonly diagnosed cancer [1]. Among HNSCCs, laryngeal squamous cell carcinoma (LSCC) is the second most common location after oral cavity squamous cell carcinoma.

Despite improvements in the field of surgery, chemotherapy, and radiotherapy, patients with LSCC still face an unfavorable prognosis. Effective therapeutic strategies and increased understanding of the relevant mechanisms implicated in treatment failure are thus needed to improve the prognosis of patients with LSCC [2].

Tumor progression and response to treatment are influenced by the interaction between tumor cells and the surrounding tumor microenvironment (TME). The TME is composed of a variety of cellular entities including fibroblasts, endothelial cells, blood vessels, lymph vessels, and cells of the immune system [3].

Evasion of the immune system is an important hallmark of cancer and various mechanisms of immune evasion have been characterized and offer key targets for anti-cancer immunotherapy [4]. It is generally accepted that tumor progression is the consequence of an imbalance between an aggressive tumor growth and host immunity, often indicated through infiltration of immune cells in the TME, purportedly to protect against tumor development [4,5,6]. Tumor-infiltrating lymphocytes (TIL) are considered the most crucial effectors of the host anti-tumor immune response, and the assessment of the degree of their infiltration has been demonstrated to be of prognostic value in various tumors [7]. In colon cancer, the Immunoscore, based on quantification of different types of T cells (CD3 and CD8-positive T cells), proved to be of greater prognostic significance than conventional TNM staging [6,8].

Similarly, the presence of TIL is increasingly recognized as an important biomarker in HNSCC and appears to have prognostic value [9,10,11]. However, the main limitation of most studies is the heterogeneity due to the inclusion of multiple tumor subsites. The prognostic value of TIL infiltration is likely to differ between different tumor subsites, but the small number of patients included in most studies do not allow for stratification based on subsites [10,11,12]. Focusing on more homogeneous patient cohorts could strengthen the conclusions of TIL as a potential prognostic biomarker and provide more insight into the differences between patient subgroups.

Although there is an increasing number of studies reporting a better prognosis in patients with high TIL in the tumor microenvironment of LSCC, these studies usually include a limited number of patients and there is no consensus on the methods for quantification of TIL and TIL analysis. Thus, routine assessment of TIL has not yet found its way to daily clinical practice [13]. Therefore, the aim of this study is to review the existing literature to analyze the role of TIL specifically in patients with LSCC.

## 2. Materials and Methods

**Figure 1:**
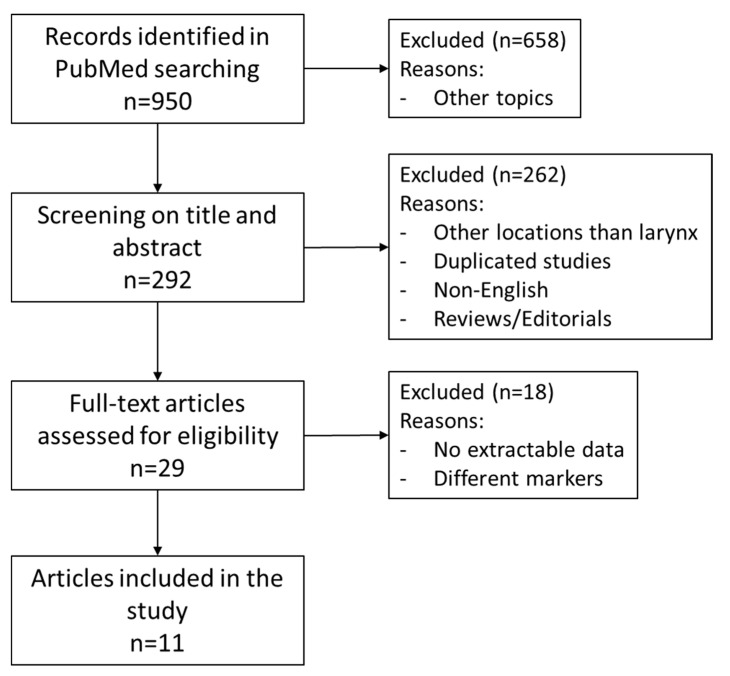
Flow chart showing the process of the study selections for the systematic review.

The Preferred Reporting Items for Systematic Review and Meta-Analyses (PRISMA) were used to conduct a systematic review of the current literature [14]. The search strategy aimed to include all articles concerning the role of TIL in LSCC. A PubMed internet search updated to 20 January 2021 was performed for English language publications between the years 1990 and 2021 using the following search criteria in the title or abstract: “tumor-infiltrating lymphocytes” coupled with “Head and Neck Squamous Cell Carcinoma” OR “Larynx Squamous Cell Carcinoma” OR “Head and neck cancer” OR “Laryngeal cancer” (Supplementary Table S1). The search results were reviewed by 2 independent researchers (JPR and MSC) for potentially eligible studies. When there was any statement in the abstract on follow-up data and outcomes of the role of TIL in LSCC, the full text article was searched; all review articles were also checked in full. References from any full text articles were cross-checked to ensure inclusion in this review if appropriate (Figure 1). Disagreements over the eligibility of an article were resolved by consensus.

Studies were selected if they met the following inclusion criteria: (1) prognostic value of TIL was evaluated in patients with LSCC (primary or recurrent), (2) TIL were evaluated either immunohistochemically (CD8+, CD4+, and/or CD3+) and/or in hematoxylin- and eosin (HE)-stained sections, in resected specimens or in diagnostic biopsies, (3) TIL were evaluated either in tumor epithelium and/or tumor stroma, (4) prognostic value of TIL was evaluated by time-to-event survival analysis with overall survival (OS) and/or disease-free survival (DFS), and (5) original articles published in English. Studies reported in reviews, letters, or conference abstracts were excluded. Studies containing aggregated data or duplicated data from previously published work were also excluded.

For these studies, a meta-analysis was undertaken using Review Manager 5.4. We conducted subgroup analysis stratified by evaluation of TIL (immunohistochemically or HE-stained sections). Heterogeneity was assessed using I^2^ statistic. Hazard ratios (HRs) were used that described the risk of event for high TIL versus low TIL. Then, we used the fixed effect model or the random effect model for the analysis. Forest plots and funnel plots were employed to test the overall effect and the publication bias, respectively. All tests were two-sided with a significance level of *p* < 0.05.

## 3. Results

According to our search criteria, 950 papers were initially identified but as could be deduced from the title, only 292 were related to the topic of the study. After sorting and removal of duplicates, all the remaining abstracts were reviewed, and 29 papers were retrieved and reviewed in detail [9,12,15,16,17,18,19,20,21,22,23,24,25,26,27,28,29,30,31,32,33,34,35,36,37,38,39,40,41,42]. The studies that did not fulfill all the inclusion criteria were discarded. Of these, seven were excluded because they did not report the outcome of LSCC separately from other locations of HNSCC [12,15,16,17,18,19,20], eight because no HRs were available [21,22,23,24,25,26,27,28,29], one study because they reported outcome in terms of local control [30], one study that evaluated Il-12R expression on tumor cells [31], and one study that evaluated lymphocytes in peripheral blood [32]. This left 11 studies for the analysis (Figure 1) [9,33,34,35,36,37,38,39,40,41,42]. All the studies included in this review were retrospective. Table 1 summarizes the main features of the selected studies and Table 2 summarizes the key findings of the selected studies.

**Figure 2:**
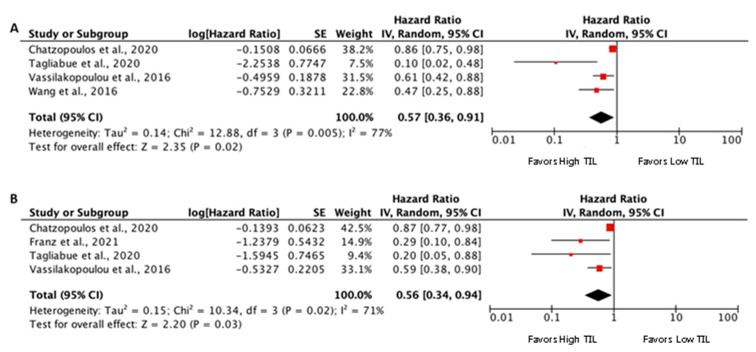
Forest plots of prognostic value of stromal TIL on hematoxylin-eosin-stained sections on overall survival (**A**) and disease-free survival (**B**).

The prognostic value of TILs in HE-stained sections was assessed in five eligible studies for inclusion in the meta-analysis [33,34,38,40,42], although not all the studies included the results of DFS and OS (Figure 2). These five studies analyzed the infiltration by TIL of the tumor stroma in resected specimens. All the studies showed a survival advantage for the cases with high TIL infiltration. The pooled meta-analysis showed an advantage for high stromal TIL (pooled HR 0.57 (95% CI 0.36–0.91), *p* = 0.02, for OS, and 0.56 (95% CI 0.34–0.94), *p* = 0.03, for DFS). All the studies reported stromal TIL density as the ratio of the area occupied by mononuclear cell infiltrates to the entire stromal area in whole HE-stained sections avoiding intra-tumor infiltration (% TILs = area occupied by mononuclear cells in tumor stroma/total stromal area). However, not all studies used the same cut-off point for high TIL infiltration. Three studies defined a high stromal infiltration by TIL when it was higher or equal to 30% [33,34,42]. Another study defined a high stromal infiltration by TIL when it was higher or equal to 5% [40], and another one defined the stromal infiltration by TIL as a continuous variable (increments of 10%) [38].

**Figure 3:**
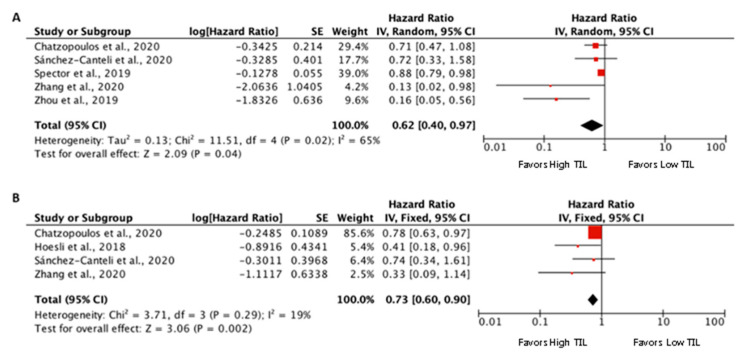
Forest plots of prognostic value of tumoral CD8+ TIL on overall survival (**A**) and disease-free survival (**B**).

The prognostic value of CD8+ TIL was assessed in six eligible studies for inclusion in the meta-analysis [9,35,37,38,39,41] (Figure 3). All the included studies showed a survival advantage for the cases with high CD8+ TIL. The pooled meta-analysis showed an advantage for high CD8+ TIL density (pooled HR 0.62 (95% CI 0.4–0.97), *p* = 0.04, for OS, and 0.73 (95% CI 0.34–0.94), *p* = 0.002, for DFS). Again, in this case the evaluation of CD8+ TIL infiltration was variable: four studies considered only tumor-infiltrating CD8+ cells (CD8+ T cells counts/mm^2^ of tumor area) and dichotomized the results by the median [9,35,38,39]; one study analyzed tumoral- (CD8+ T cells counts/mm^2^ of tumor area) and stromal (CD8+ T cells counts/mm^2^ of stromal area)-infiltrating CD8+ cells dichotomized by the median [37]; and in another one an Immunoscore was used [41], following the methodology described by Galon et al. [8], considering high infiltration a score ≥ 2.

**Figure 4:**
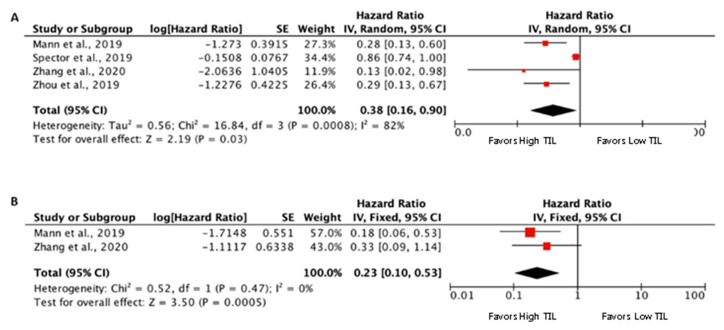
Forest plots of prognostic value of stromal and/or tumoral CD3+/CD4+ TIL on overall survival (**A**) and disease-free survival (**B**).

The prognostic value of CD4+ and/or CD3+ TIL was assessed in four eligible studies for inclusion in the meta-analysis [9,36,37,41] (Figure 4). The pooled meta-analysis showed an advantage for high CD4+ and/or CD3+ TIL (pooled HR 0.32 (95% CI 0.16–0.9), *p* = 0.03, for OS, and 0.23 (95% CI 0.10–0.53), *p* = 0.0005, for DFS). Again, the methods of evaluation were diverse. Two studies evaluated tumor-infiltrating CD4+ cells (CD4+ T cells counts/mm^2^ of tumor area) dichotomized at the median [9,36], another study stromal-infiltrating CD3+ cells (CD3+ T cells counts/mm^2^ of stromal area) dichotomized at the median [37], and in another study an Immunoscore higher or equal to 2 [41].

**Figure 5:**
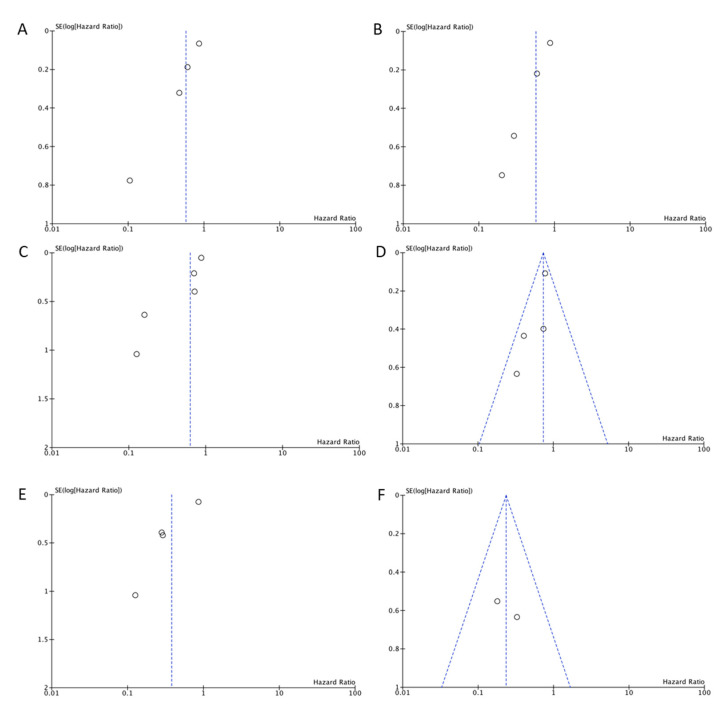
Funnel plot for assessment of potential publication bias in studies investigating the correlation between TIL infiltration evaluated in hematoxylin-eosin sections and the overall (**A**) and disease-free survival (**B**) of laryngeal cancer patients; the correlation between CD8+ TIL infiltration and the overall (**C**) and disease-free survival (**D**) of laryngeal cancer patients; and the correlation between CD3/CD4+ TIL infiltration and the overall (**E**) and disease-free survival (**F**) of laryngeal cancer patients.

The potential for publication bias was assessed by the visual inspection of funnel plots, since the number of studies for each meta-analysis was too small (less than 10) to allow the use of tests for funnel plot asymmetry. As shown in Figure 5, funnel plot asymmetry was observed on the outcomes of OS and DFS for the different methods of evaluation of TIL, suggesting the existence of a publication bias that must be considered when interpreting the results.

## 4. Discussion

In this systematic review we investigated the prognostic value of TIL in the tumor microenvironment of LSCC. We performed a meta-analysis of 11 studies with a total of 1398 patients with LSCC, in which TIL were quantified either immunohistochemically for CD8+, CD4+, and/or CD3+, or in HE-stained sections in tumor epithelium and/or tumor stroma. The major finding in this meta-analysis was the demonstration that despite different evaluations, TIL were strong prognostic factors for OS and DFS in patients with LSCC.

Previous studies that characterized immune infiltrates in the TME of HNSCC generally suggested that high levels of TIL were associated with improved patient survival [5]. Specifically, in LSCC, pioneering studies already showed that lymphoplasmacellular infiltration in the tumor stoma was correlated with a better outcome [43]. Recent meta-analyses including all HNSCC locations confirmed the favorable prognostic role of CD3+ and CD8+ T cell infiltration [10,11]. However, as highlighted by the authors of that meta-analyses, the main limitation of most studies is the heterogeneity due to tumor subsites. Previous studies with a large number of patients, that included several HNSCC subsites, confirmed that the levels of T cell infiltrates differed significantly by tumor site and tumor stage [12,39]. Therefore, we focused our analysis on a homogeneous tumor location (LSCC) with the aim to strengthen the conclusions of the study. However, it was not possible to perform an analysis by stage due to the lack of information in the studies, probably because most of them included a limited number of patients. The first and most important observation to emphasize from the analysis of the collected data in this specific subsite is the consistently better clinical outcome in terms of OS and DFS for the patients that showed a higher TIL infiltration, either evaluated in HE-stained sections or by immunohistochemistry using different markers for specific T cells (CD8+, CD3+, and CD4+). Both stromal and intra-parenchymal TIL were evaluated in the studies reviewed; however, it remains unclear as to which location of TIL has the greater prognostic significance. Future work will need to better evaluate and compare both locations in large prospective studies to answer this question. However, to incorporate TIL in clinical practice, it is also necessary to determine standardized, validated cut-offs for quantification in both HE and immunohistochemical approaches. The methods of quantifying TIL differed strongly among the studies included in this meta-analysis, and therefore it was not possible to suggest general cut-offs. An active area of research is the development of automated image analysis of HE and immunohistochemically stained whole tumor sections using sophisticated algorithms for digitized images with the addition of multiplex immunohistochemistry to simultaneously assess multiple subsets of T lymphocytes [20,44]. Using these methods, the density of TILs in different locations can be standardized and perhaps better estimates of “high” vs. “low” infiltrates can be established that could be useful in routine pathology practice.

Perhaps, the better example of a potential clinical translation of the immune contexture into a standardized prognostic marker was the establishment of the “Immunoscore” in colorectal cancer (CRC) [3,6,8]. The Immunoscore is a direct measure of T cell infiltration into tumors and is based on the quantification of CD3+ and CD8+ T cells, both at the center of tumor (CT) and at the invasive margin (IM). It provides a scoring system ranging from low immune cell densities found at both the CT and the IM (Immunoscore 0, I0), to high densities classed as Immunoscore 4 (I4), with an increasing score correlating with longer patient survival [3]. The results of our meta-analysis suggest that an immunoscore could have also prognostic significance in LSCC. In fact, a recent study using the methodology of the Immunoscore has shown that LSCC patients with an Immunoscore of 0–1 experienced the worst OS and DFS, compared with Immunoscore 2–4 [41].

The Immunoscore also has limitations. First, it is a semi-quantitative assay that needs immunohistochemistry staining of CD3+ and CD8+ T cells and digital pathology. In contrast, the optical TIL evaluation from an HE is more accessible. However, when it was compared with the Immunoscore, a 48% discordance was found, suggesting the need of digital pathology for correct and reproducible evaluation [45]. However, the results of our meta-analysis show that both the HE evaluation of TIL and the quantification of immunohistochemically stained CD8+ or CD3+/CD4+ T cells were significantly associated with DFS and OS in LSCC patients. Only one of the reviewed studies compared the evaluation of TIL in HE-stained sections and by CD8+ staining [38]. In this study, both high TIL density in HE sections and high CD8+ counts were associated with a lower risk of relapse and death. However, TIL density and CD8+ counts weakly correlated with each other, and in the multivariate analysis only TIL density was independently associated with survival and recurrence risk. This suggests that assessment of CD8+ infiltrates does not seem to offer additional prognostic information over the morphologically assessed TIL density in LSCC [38]. A simple and cost-effective method for the assessment of TIL in HE-stained sections in different cancers was proposed by the International Immuno Oncology Biomarkers Working Group in 2017 [46,47], with a high inter-observer agreement. Briefly, in this method, TIL (all mononuclear cells, including lymphocytes and plasma cells) should be reported separately for the stromal compartment (% stromal TIL) and the tumor cell compartment (% intra-tumoral TIL), and should be evaluated within the borders of the invasive tumor, including both “central tumor” and “invasive margin”. One section (magnification 200–400×) per patient can be considered to be sufficient for practical purposes, and full sections are preferred over biopsies whenever possible. A full assessment of average TIL in the tumor area (central tumor and invasive margin) should be used and TIL should be assessed as a continuous variable. Until now, no formal recommendation for a clinically relevant TIL threshold(s) has been established [46]. This method has shown prognostic significance in different HNSCC sites with good reproducibility [48]. However, this proposal does not have any clinical application yet and validating studies for each subsite of HNSCC are required to obtain a more definitive conclusion on its applicability and the most optimal cut-off point.

Another limitation of the Immunoscore is that it is designed for its use in resected tumors because it evaluates the tumor core and the invasive margin, and the latter is rarely available in incisional biopsies. Another problem in assessing TIL in biopsies is immune response heterogeneity, as the TIL infiltration in tumor is not homogenous. All the studies included in our meta-analysis that evaluated TIL infiltration in HE sections were in whole FFPE sections of resected specimens, and the quantification was in the stroma. The studies that evaluated CD3+, CD4+, and/or CD8+ TIL were also all performed in resected specimens, except for one [9], although most of them analyzed TIL density in tissue microarrays. Interestingly, the study of Spector et al. [9] also found a significantly improved OS for patients with higher CD8+ counts and a higher TIL-weighted score (a combination of CD8+, CD4+, and FOXP3+ TIL). Similarly, Balermpas et al. [17], in a study including different HNSCC locations (but only seven LSCC) found that a high CD3+ and CD8+ expression in pretreatment biopsies of patients that received definitive chemoradiotherapy was associated with a higher OS and DFS. The results of these studies suggest that TIL density could be also evaluated in diagnostic biopsies and this evaluation could have the same prognostic importance as that of the evaluation obtained from resected specimens. In this way, an Immunoscore for biopsy (without the need of the invasive margin for quantification) has been developed for CRC and compared with the consensus Immunoscore for resected tumors. This Immunoscore on biopsy also significantly predicts the prognosis of the patients, even if not as powerful as the Immunoscore incorporating the invasive margin [49].

Another issue to be considered is the treatment modality. Since different treatment modalities have a different mechanism of action, the prognostic value of TIL could also depend on the given therapy [12]. As all but one of the studies included in our meta-analysis were performed in resected specimens, we can only conclude on the general prognostic value of T cell markers in surgically treated patients, while the predictive value for other treatment modalities must be elucidated. To incorporate TIL as prognostic markers in clinical practice in LSCC, large prospective studies with uniform methodology and homogeneous patient cohorts with respect to tumor subsite and treatment modality are needed that can consider traditional clinical and tumor variables and newer variables such as human papilloma virus, immune-related gene expression, and perhaps other tumor genetics.

This study also has several limitations. In addition to the retrospective nature of all the studies, the main weakness of this review is that the number of patients enrolled in the different studies included in this analysis was quite small: only five of 11 studies included more than 100 patients. Moreover, there was not a validated cut-off for TIL quantification. This heterogeneity could also have influenced the results, since not all the TIL were comparable. It must be considered also the possible existence of a publication bias, as can be deduced from the examination of funnel plots.

## 5. Conclusions

In conclusion, this meta-analysis confirmed the prognostic significance of TIL in the largest reported cohort meta-analysis of LSCC. Immune infiltrates in the TME appeared consistently important regardless of method of assessment. High stromal TIL evaluated in HE-stained sections and intra-tumoral and stromal CD3+, CD4+, and/or CD8+ TIL predicted a better clinical outcome. Based on these data, the assessment of TIL in the routine pathology report for patients with LSCC using either HE-stained tumor sections or immunohistochemistry deserves further study as a standardized and validated biomarker.

## Figures and Tables

**Table 1:** Main features of the selected studies.

References	Analytic Cohort	Covariant	Sample	Stage	Treatment	Technique	Other Biomarkers
Vassilakopoulou et al., 2016 [33]	260	High stromal TIL (≥30%)	Postoperative sample	All	S, S+RT	HE in WS	PD-L1 (IHC and mRNA)
Wang et al., 2016 [34]	120	High stromal TIL (≥30%)	Postoperative sample	All	S+RT, S+CRT	HE in WS	No
Hoesli et al., 2018 [35]	183	High tumor-infiltrating CD8+ (dichotomized at the median)	Postoperative sample	All	S (post RT, CRT)	IHC in TMAs	PD-L1 CPS (IHC)
Mann et al., 2019 [36]	183	High tumor-infiltrating CD103+ and CD4+	Postoperative sample	All	S (post RT, CRT)	IHC in TMAs	No
Spector et al., 2019 [9]	74	High tumor-infiltrating CD8+ (dichotomized at the median) and high tumoral TILws (0.35 × CD8+ 0.35 × CD4+ 0.3 × FOXP3+)	Incisional biopsies	All	S, S+(C)RT, RT, CRT	IHC in TMAs	No
Zhou et al., 2019 [37]	71	High tumor- and stromal-infiltrating CD8+ and high stromal-infiltrating CD3+ (dichotomized at the median)	Postoperative sample	All	S	IHC in WS	CD163 (IHC)
Chatzopoulos et al., 2020 [38]	283	Stromal TIL (10% increments) and high tumor-infiltrating CD8+ (dichotomized at the median)	Postoperative sample	All	S, S+RT	HE in WS and IHC in TMAs	No
Sánchez-Canteli et al., 2020 [39]	67	High tumor-infiltrating CD8+ (dichotomized at the median)	Postoperative sample	All	S, S+RT	IHC in TMAs	PD-L1 CPS and TPS (IHC)
Tagliabue et al., 2020 [40]	56	High stromal TIL (≥5%)	Postoperative sample	III/IV	S, S+(C)RT, RT, CRT	HE in WS	No
Zhang et al., 2020 [41]	41	Immunoscore (2–4) vs (0–1) (CD3+/CD8+)	Postoperative sample	All	S	IHC in WS	CD66b+ (IHC)
Franz et al., 2021 [42]	60	High stromal TIL (≥30%)	Postoperative sample	All	S, S+RT	HE in WS	PD-L1 CPS (IHC) NLR

TIL: tumor-infiltrating lymphocytes; S: surgery; RT: radiotherapy; CT: chemotherapy; CRT: chemoradiotherapy; HE: hematoxylin-eosin; IHC: immunohistochemistry; NLR: neutrophil-to-lymphocyte ratio; TMAs: tissue microarrays; WS: whole sections.

**Table 2:** Key findings of the selected studies.

References	Key Findings
Vassilakopoulou et al., 2016 [33]	Increased TIL density in tumor stroma was associated with better outcome in laryngeal squamous cell carcinoma.
Wang et al., 2016 [34]	High TIL density in tumor stroma, which represents the local inflammation, was predictive of longer OS and RFS.
Hoesli et al., 2018 [35]	CD8+ TIL status was associated with a significant improvement in DFS and DSS in patients with recurrent/persistent laryngeal squamous cell carcinoma surgically treated after RT/CRT.
Mann et al., 2019 [36]	An immune profile driven by CD103+ TIL content, alone and in combination with CD4+ TIL content, was a prognostic biomarker of survival in patients with recurrent/persistent LSCC.
Spector et al., 2019 [9]	The levels of TIL were an independent prognostic factor in patients with head and neck squamous cell carcinoma, including laryngeal squamous cell carcinoma, treated with different modalities. Subsets of TILs and combined TILs scores may be clinically useful predictive and prognostic factors.
Zhou et al., 2019 [37]	High density of peritumoral CD3+ and CD8+ immune cells in both tumor stroma and tumor nests was significantly associated with a favorable OS.
Chatzopoulos et al., 2020 [38]	Favorable prognostic impact of higher TIL density in laryngeal squamous cell carcinoma patients. The assessment of CD8+ infiltrates does not seem to offer additional prognostic information over the morphologically assessed TIL density.
Sánchez-Canteli et al., 2020 [39]	High tumoral infiltration by CD8+ TIL was associated with better survival outcomes.
Tagliabue et al., 2020 [40]	Low TIL and altered expression of specific genes associated with tumor–immune systems interactions emerged as independent risk factors, associated with poor prognosis and relapse within 2 years in advanced laryngeal squamous cell carcinoma.
Zhang et al., 2020 [41]	High density of CD3+ TIL were associated with better OS. Patients with an Immunoscore of 0–1 experienced the worst OS and DFS, compared with Immunoscore 2–4.
Franz et al., 2021 [42]	TIL count rate ≥30% was associated with higher DFS and reduced recurrence risk.

OS: overall survival; DSS: disease-specific survival; DFS: disease-free survival; RFS: recurrence-free survival; RT: radiotherapy; CRT: chemoradiotherapy.

## Data Availability

No new data were created or analyzed in this study. Data sharing is not applicable to this article.

## Supplementary Materials

The following are available online at https://www.mdpi.com/article/10.3390/biomedicines9050486/s1. Table S1: Search strategy in PubMed.
