# Supplementary material for: Tumor-Infiltrating Lymphocytes in the Tumor Microenvironment of Laryngeal Squamous Cell Carcinoma: Systematic Review and Meta-Analysis
Source: Biomedicines. 2021 Apr 28;9(5):486. doi: 10.3390/biomedicines9050486 (PMC8145951; doi:10.3390/biomedicines9050486)
Supplement: Supplementary file 1 [file biomedicines-09-00486-s001.zip › biomedicines-1163179-supplementary.pdf]

SUPPLEMENTARY MATERIAL

Supplementary Table 1: Search strategy in PubMed

**Search:**<br>**(tumor-infiltrating lymphocytes) AND ((((head and neck squamous cell carcinoma) OR (head and neck cancer) OR (larynx squamous cell carcinoma) OR (laryngeal cancer))))**<br>("tumour infiltrating lymphocytes"[All Fields] OR "lymphocytes, tumor infiltrating"[MeSH Terms] OR ("lymphocytes"[All Fields] AND "tumor infiltrating"[All Fields]) OR "tumor-infiltrating lymphocytes"[All Fields] OR ("tumor"[All Fields] AND "infiltrating"[All Fields] AND "lymphocytes"[All Fields]) OR "tumor infiltrating lymphocytes"[All Fields]) AND ("squamous cell carcinoma of head and neck"[MeSH Terms] OR ("squamous"[All Fields] AND "cell"[All Fields] AND "carcinoma"[All Fields] AND "head"[All Fields] AND "neck"[All Fields]) OR "squamous cell carcinoma of head and neck"[All Fields] OR ("head"[All Fields] AND "neck"[All Fields] AND "squamous"[All Fields] AND "cell"[All Fields] AND "carcinoma"[All Fields]) OR "head and neck squamous cell carcinoma"[All Fields] OR ("head and neck neoplasms"[MeSH Terms] OR ("head"[All Fields] AND "neck"[All Fields] AND "neoplasms"[All Fields]) OR "head and neck neoplasms"[All Fields] OR ("head"[All Fields] AND "neck"[All Fields] AND "cancer"[All Fields]) OR "head and neck cancer"[All Fields]) OR ("squamous cell carcinoma of head and neck"[MeSH Terms] OR ("squamous"[All Fields] AND "cell"[All Fields] AND "carcinoma"[All Fields] AND "head"[All Fields] AND "neck"[All Fields]) OR "squamous cell carcinoma of head and neck"[All Fields] OR ("larynx"[All Fields] AND "squamous"[All Fields] AND "cell"[All Fields] AND "carcinoma"[All Fields]) OR "larynx squamous cell carcinoma"[All Fields]) OR ("laryngeal neoplasms"[MeSH Terms] OR ("laryngeal"[All Fields] AND "neoplasms"[All Fields]) OR "laryngeal neoplasms"[All Fields] OR ("laryngeal"[All Fields] AND "cancer"[All Fields]) OR "laryngeal cancer"[All Fields]))
**Translations**<br>**tumor-infiltrating lymphocytes:** "tumour infiltrating lymphocytes"[All Fields] OR "lymphocytes, tumor-infiltrating"[MeSH Terms] OR ("lymphocytes"[All Fields] AND "tumor-infiltrating"[All Fields]) OR "tumor-infiltrating lymphocytes"[All Fields] OR ("tumor"[All Fields] AND "infiltrating"[All Fields] AND "lymphocytes"[All Fields]) OR "tumor infiltrating lymphocytes"[All Fields]<br>**head and neck squamous cell carcinoma:** "squamous cell carcinoma of head and neck"[MeSH Terms] OR ("squamous"[All Fields] AND "cell"[All Fields] AND "carcinoma"[All Fields] AND "head"[All Fields] AND "neck"[All Fields]) OR "squamous cell carcinoma of head and neck"[All Fields] OR ("head"[All Fields] AND "neck"[All Fields] AND "squamous"[All Fields] AND "cell"[All Fields] AND "carcinoma"[All Fields]) OR "head and neck squamous cell carcinoma"[All Fields]<br>**head and neck cancer:** "head and neck neoplasms"[MeSH Terms] OR ("head"[All Fields] AND "neck"[All Fields] AND "neoplasms"[All Fields]) OR "head and neck neoplasms"[All Fields] OR ("head"[All Fields] AND "neck"[All Fields] AND "cancer"[All Fields]) OR "head and neck cancer"[All Fields]<br>**larynx squamous cell carcinoma:** "squamous cell carcinoma of head and neck"[MeSH Terms] OR ("squamous"[All Fields] AND "cell"[All Fields] AND "carcinoma"[All Fields] AND "head"[All Fields] AND "neck"[All Fields]) OR "squamous cell carcinoma of head and neck"[All Fields] OR ("larynx"[All Fields] AND "squamous"[All Fields] AND "cell"[All Fields] AND "carcinoma"[All Fields]) OR "larynx squamous cell carcinoma"[All Fields]<br>**laryngeal cancer:** "laryngeal neoplasms"[MeSH Terms] OR ("laryngeal"[All Fields] AND "neoplasms"[All Fields]) OR "laryngeal neoplasms"[All Fields] OR ("laryngeal"[All Fields] AND "cancer"[All Fields]) OR "laryngeal cancer"[All Fields]
**Results: N=950**
